# Effect of the kinematic retaining design on knee kinematics in total knee arthroplasty: A cadaveric study using a navigation system

**DOI:** 10.1186/s43019-025-00290-5

**Published:** 2025-09-16

**Authors:** Tomofumi Kinoshita, Kazunori Hino, Tatsuhiko Kutsuna, Kunihiko Watamori, Takashi Tsuda, Yusuke Horita, Masaki Takao

**Affiliations:** 1 Department of Orthopaedic Surgery, Ehime University Graduate School of Medicine, Toon, Ehime Japan; 2 Department of Joint Reconstruction, Ehime University Graduate School of Medicine, Toon, Ehime, Japan

**Keywords:** Knee kinematics, Total knee arthroplasty, Medial pivot, Kinematic retaining, Cadaver study

## Abstract

**Background:**

Implant design in total knee arthroplasty (TKA) has evolved considerably, with recent developments focusing on reproducing native knee kinematics. Some implants now feature anatomically and physiologically accurate articular surface geometries. This study aimed to evaluate the impact of different implant designs on knee kinematics using the same cadaveric specimens to ensure consistent comparison. We hypothesized that implant designs incorporating features intended to replicate native joint anatomy, such as the kinematic retaining (KR) design, would more closely reproduce physiological knee kinematics.

**Methods:**

TKA was performed on nine Thiel-embalmed cadaveric knees with mild medial osteoarthritis, using three implant designs from the Physica system: KR, cruciate retaining (CR), and medial congruent (MC) designs. All procedures were performed using a mechanical alignment technique, with both the posterior tibial slope and femoral rotational angle standardized at 3°. The posterior cruciate ligament was preserved throughout the evaluation of all implant designs. A navigation system was used to collect detailed kinematic data. Evaluations were conducted after trial component placement, focusing on anteroposterior, mediolateral, and compression–distraction positions, as well as rotational angles. From these knee status data, femoral rotational kinematics relative to the tibia and the anteroposterior translation of both femoral condyles during flexion were also calculated.

**Results:**

No significant differences in flexion and extension angles were observed between the groups. The KR group presented the greatest mean femoral external rotation relative to the tibia throughout the range of motion among the groups; however, there were no statistically significant differences. The CR and MC group showed significantly reduced anteroposterior translation of the lateral condyle compared with the native knee (*p* = 0.021 and 0.003, respectively). Furthermore, the anteroposterior translation of the lateral femoral condyle was significantly greater in the KR group than in MC groups (*p* = 0.021). In the KR group, six of nine knees exhibited medial pivot motion, compared with three in the CR group and four in the MC group.

**Conclusions:**

Using identical cadaveric specimens and navigation-based analysis, we identified distinct kinematic profiles associated with each implant design. Notably, the KR implant demonstrated kinematics approximating native knee motion; however, these findings remain preliminary and warrant further clinical validation.

**Level of Evidence:**

III.

**Supplementary Information:**

The online version contains supplementary material available at 10.1186/s43019-025-00290-5.

## Background

Restoring normal knee kinematics after total knee arthroplasty (TKA) remains a key challenge for both surgeons and implant designers. Recent advances in evaluation technologies have revealed that various factors influence knee kinematics [1–3]. One such factor is alignment: Preoperative alignment has been shown to affect preoperative kinematics [1], and preoperative kinematic patterns may resemble those observed postoperatively [2]. In addition, the intraoperative alignment strategy is also considered a factor related to kinematic outcomes [3]. Nevertheless, numerous studies have reported substantial deviations from native knee motion following TKA [4]. Nishio et al. suggested that achieving more natural kinematics may improve postoperative outcomes [5]. Although a new implant design as bicruciate-retaining TKA (BCR-TKA)attempted to preserve ligament balance by retaining the anterior cruciate ligament (ACL), some research demonstrated the difficulty to fully replicate normal knee motion and high revision rates [6, 7]. In addition, ACL function is often lost in end-stage knee osteoarthritis (KOA) [8], further complicating restoration efforts and driving ongoing implant development.

Bicruciate-stabilized (BCS) TKA was introduced to compensate for both the ACL and posterior cruciate ligament (PCL) using a cam–post mechanism. However, its kinematics differ from those of the native knee [4]. Similarly, medial pivot-type inserts, designed to guide physiological rotation, exhibit deviations from normal motion [9]. These inconsistencies may be partly due to varying preoperative conditions, making comparisons across patient populations challenging. Therefore, a new implant concept called kinematic retaining (KR) was proposed. The KR femoral component features thicker medial, distal, and lateral posterior condyles, whereas its insert mimics natural joint anatomy with a 3° varus joint line, a 3° posterior slope, and convex lateral and concave medial surfaces. This design aimed to maintain a consistent tibiofemoral gap throughout the range of motion while preserving the PCL. However, the kinematic validation of this implant remains limited.

This study aimed to assess kinematic differences using a navigation system by performing TKA with three different implant designs on the same cadaveric knee under identical surgical procedures and alignments. We hypothesized that the KR design would more accurately reproduce normal knee kinematics in TKA.

## Methods

### Study aim

This study aimed to evaluate the impact of different implant designs on knee kinematics using the same cadaveric specimens for consistent comparison with a navigation system.

### Study setting

This study was conducted in a controlled laboratory setting using Thiel-embalmed cadaveric knees.

This study adhered to the principles outlined in the Declaration of Helsinki and was approved by the institutional review board. TKA using the Physica System (Enovis, Limacorporate S.p.A, Udine, Italy) was performed on nine knees from five Thiel-embalmed cadavers (three males and two females). The cadavers had a mean age of 84.7 years (standard deviation [SD] ± 5.1, range 75–94 years) at the time of death. None of the patients had a history of prior knee surgeries or exhibited macroscopic signs of traumatic or advanced degenerative changes. All specimens presented with only mild osteoarthritic (OA) alterations.

### Registration of the navigation system

A navigation system (version 4.0, Precision Knee Navigation Software; Stryker, Kalamazoo, MI, USA) was used to assess intraoperative knee kinematics. Infrared trackers were affixed to the femur and tibia using pins to detect specific anatomical landmarks. A skin incision was made to access the subcutaneous layers, and registration was performed using both the osteophytes and surrounding soft tissue structures while preserving the ACL. The rotational axes of the femur and tibia were determined on the basis of anatomical references. The femoral rotational axis was defined as perpendicular to the Whiteside’s line and parallel to the transepicondylar axis. On the tibial side, the rotational axis was aligned parallel to the line extending from the medial one-third of the tibial tubercle to the midpoint of the transverse diameter.

### Preoperative evaluation of knee status using the navigation system

Once the registration was completed, the joint capsule was temporarily approximated using four sutures. Subsequently, passive knee flexion was performed manually in a smooth motion, from full extension to deep flexion, avoiding any angular acceleration. The navigation system automatically recorded measurements of anteroposterior (AP), mediolateral, compression–distraction positions, and internal–external rotation angles of the tibia relative to the femur at knee flexion angles of 0°, 5°, 10°, 15°, 20°, 30°, 45°, 60°, 90°, 105°, and 120°, as well as at the maximum flexion angle achieved. In cases where the full extension was restricted by flexion contracture, data collection began from the maximum achievable extension angle and continued through the predefined flexion points. For the AP position, positive values indicated an anterior position, whereas negative values indicated a posterior position of the tibia relative to the femur. For the mediolateral position, positive values indicated a medial position, whereas negative values indicated a lateral position of the tibia relative to the femur. For the compression–distraction position, positive values indicated compression, whereas negative values indicated a distraction of the tibia relative to the femur. For the rotational angle, positive values indicated internal rotation, whereas negative values indicated external rotation of the tibia relative to the femur. Measurements were captured at increments of 0.5° or 1 mm. An experienced orthopedic surgeon performed all procedures to ensure consistency. To verify the accuracy and reproducibility of the navigation-based measurements, we assessed test–retest reliability. At each recorded knee-flexion angle, both the inter-and intraclass correlation coefficients exceeded 0.9, indicating excellent reliability. This high level of reproducibility was observed across all measured parameters, including the AP, mediolateral, and compression–distraction positions, as well as the rotational angles. To evaluate the AP position of the femur relative to the tibia, we calculated the femoral center movement using a previously established method [1, 10]. This was derived from the AP and compression–distraction positions of the tibia relative to the femur, as obtained using the navigation system.

### Surgical procedure

For the surgical procedure, a measured resection technique was applied, with the femoral component positioned at an external rotation of 3° relative to the posterior condylar axis. Distal femoral and proximal tibial resections were performed perpendicular to their respective mechanical axes in alignment with the mechanical alignment concept. The femoral sagittal resection angle and posterior tibial slope were standardized at 0° and 3°, respectively, under navigation system guidance in all cases. Following the removal of osteophytes, trial components and inserts were placed using a 10-mm insert, which is the minimum available thickness for this TKA system. Then, the joint capsule was temporarily closed. Subsequently, we re-evaluated the AP and mediolateral positions, as well as internal–external rotation angles, at maximum extension and knee flexion angles of 0°, 5°, 10°, 15°, 20°, 30°, 45°, 60°, 90°, 105°, 120°, and the maximum flexion angle using the same measurement procedures employed preoperatively. In cases where the full extension was limited owing to flexion contracture, data were recorded from the maximum achievable extension angle to maximum flexion. Using this method, three different implant designs were implanted sequentially in the following order: (1) KR, (2) cruciate retaining (CR), and (3) medial congruent (MC). For each design, the knee-joint status was recorded using the navigation system. The PCL was preserved throughout the evaluation of all implant designs. Furthermore, no additional bone resections of the femur or tibia were performed, and no supplementary soft tissue releases, including those involving the medial collateral ligament, were required.

**Fig. 1:**
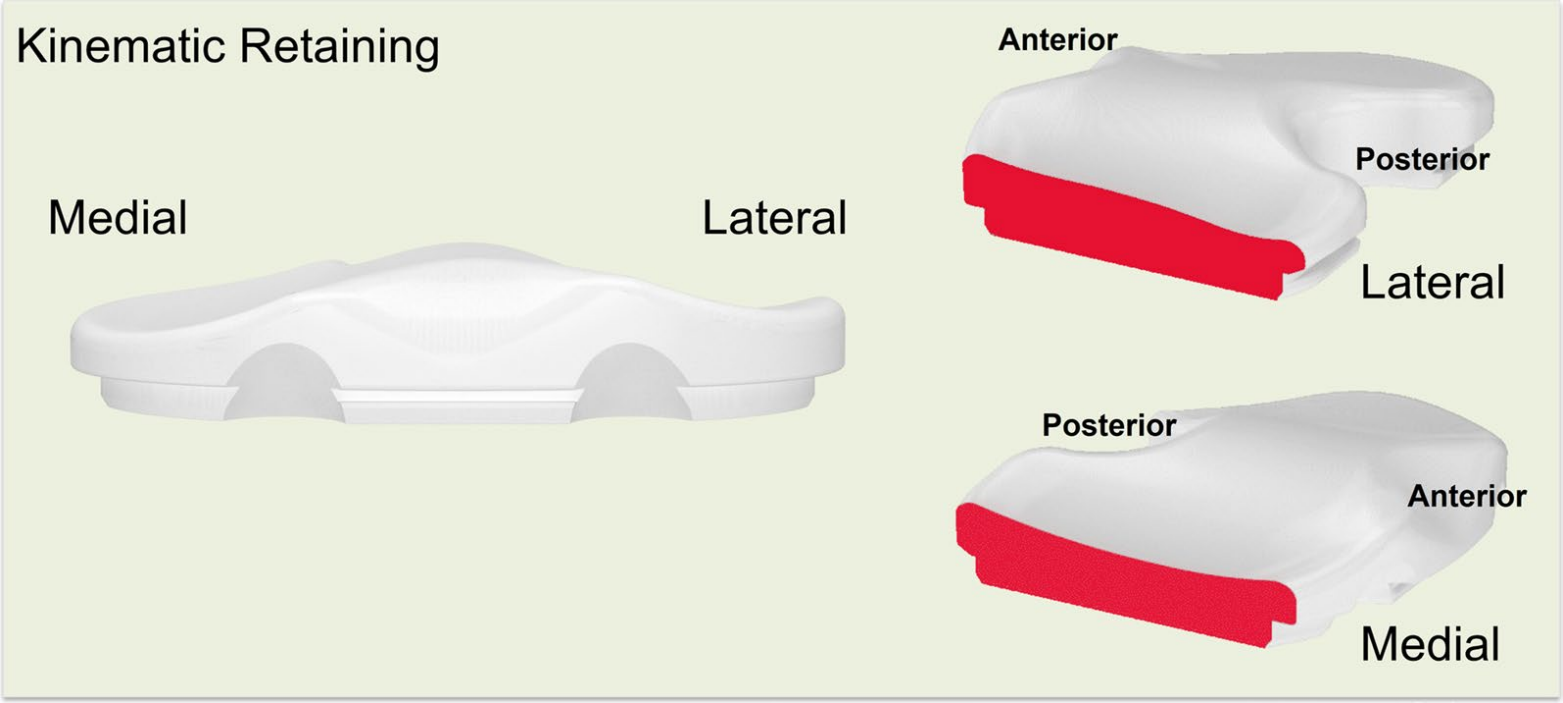
Illustration of kinematic-retaining design insert. The figure on the left shows the anterior view of the kinematic retaining design insert, featuring a 0° slope on the tibial insert and a built-in 3° varus joint line inclination. The figure on the right illustrates the difference in the insert shape between the lateral and medial sides

**Fig. 2:**
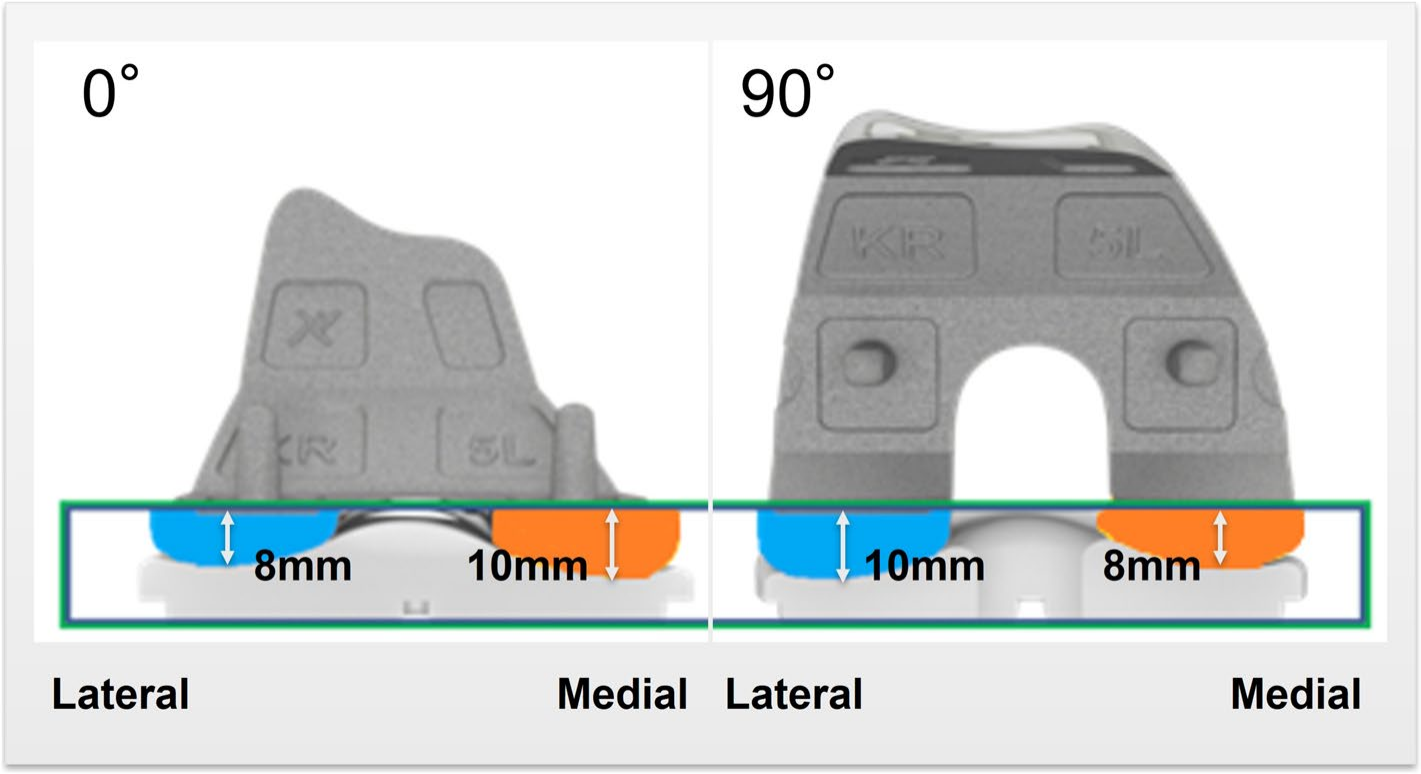
Illustration of kinematic-retaining design of femoral component. The thickness of the femoral component varies between the distal to the posterior condyles to maintain a consistent, rectangular joint space. Specifically, the KR femoral component is thicker medially and thinner laterally from a distal perspective, whereas from a posterior view, it is thicker laterally and thinner medially

**Fig. 3:**
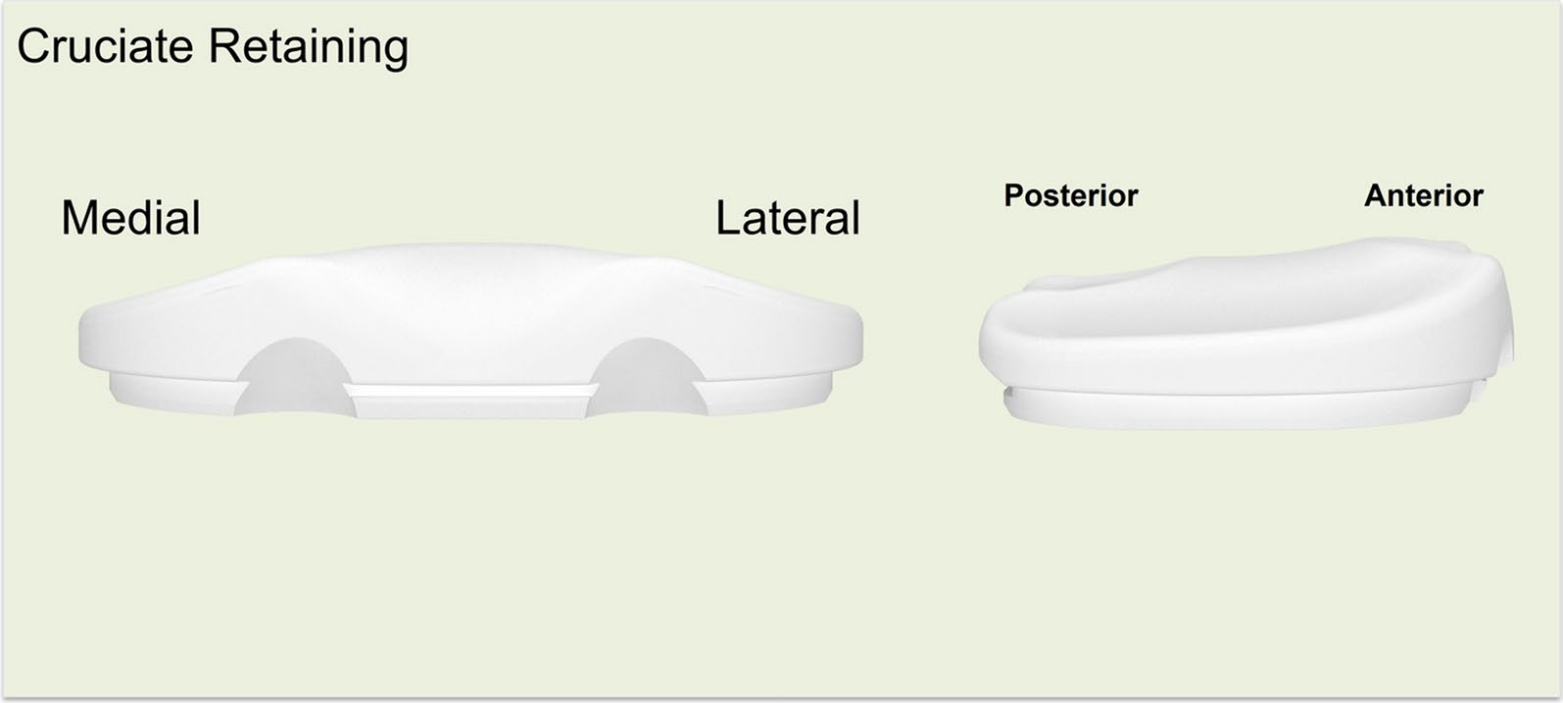
Illustration of cruciate-retaining design insert. The figure on the left shows the anterior view of the cruciate-retaining design insert, whereas the figure on the right shows the lateral view. The articulating surface of the cruciate-retaining insert features a concave shape in both the lateral and medial compartments

**Fig. 4:**
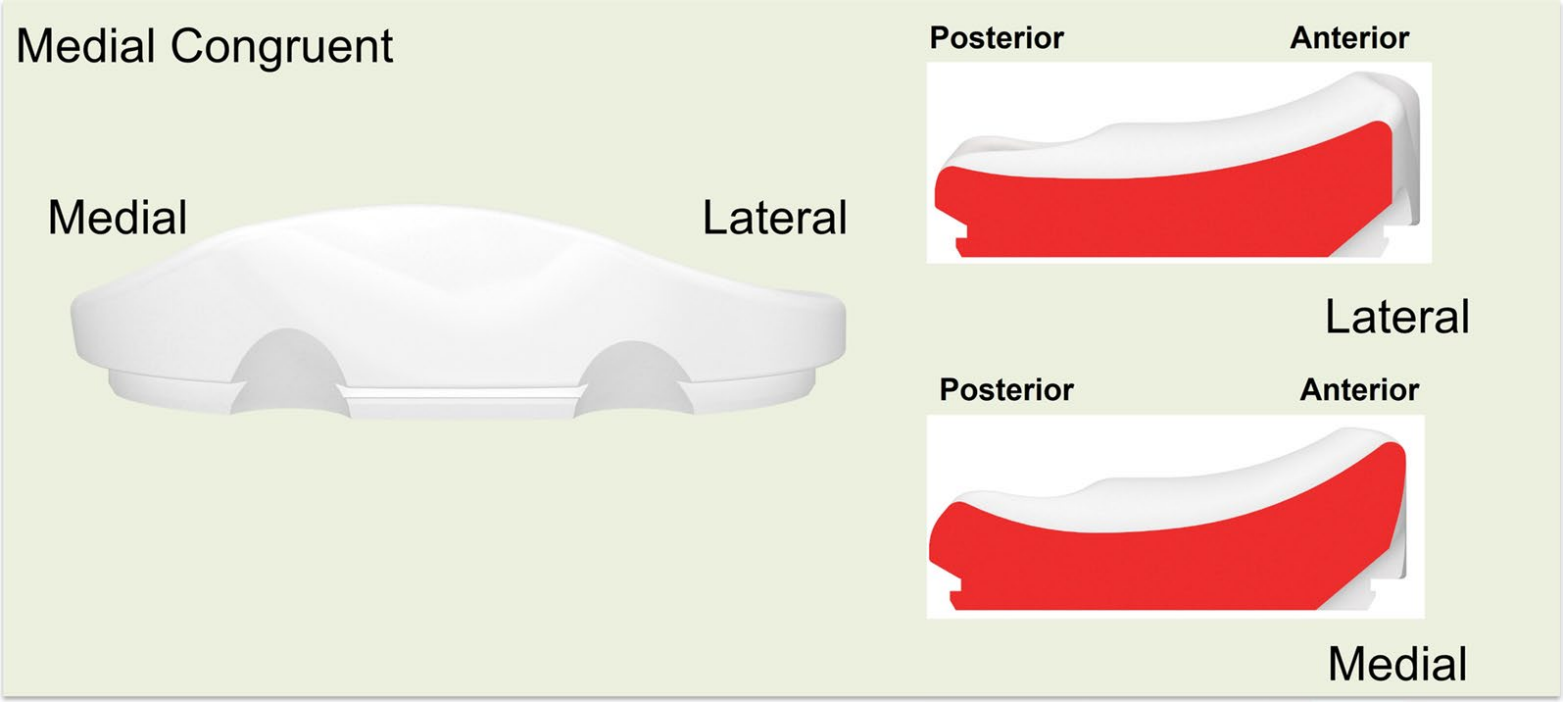
Illustration of medial congruent design insert. The figure on the left shows the anterior view of the medial congruent design insert. The tibial insert has an asymmetric configuration that reflects the different shapes of the medial and lateral tibial plateaus of a normal knee. The figure on the right illustrates the difference in the insert shape between the medial and lateral sides

With respect to the insert and implant design, the KR design features a lateral tibial plateau that mimics the native tibia, exhibiting a convex in the AP direction. It included the anterior and posterior lips to prevent excessive translation due to the absence of the ACL and meniscus. The medial tibial plateau was concave in the AP direction, similar to the native tibial anatomy, with a 3° slope in the tibial liner and a built-in varus joint line inclination of 3° (Fig. 1). The thickness of the femoral component varies from the distal to the posterior condyles to maintain a consistent rectangular joint space. Specifically, the KR femoral component was thicker medially and thinner laterally from a distal perspective, whereas from a posterior view, it was thicker laterally and thinner medially (Fig. 2). In the CR design, the articulating surface features a concave shape in both the lateral and medial compartments (Fig. 3). The MC design incorporates an asymmetric tibial insert that reflects the different shapes of the medial and lateral tibial plateaus. The medial plateau features deeper concavity, providing greater congruency and anterior constraint, thereby stabilizing the knee from full extension through deep flexion. In contrast, the lateral plateau was designed to allow the lateral femoral condyle to move freely along an open path, replicating the motion of a healthy knee (Fig. 4). The lateral tibial plateau shares the same geometry as the CR design.

### Kinematic evaluation method

**Fig. 5:**
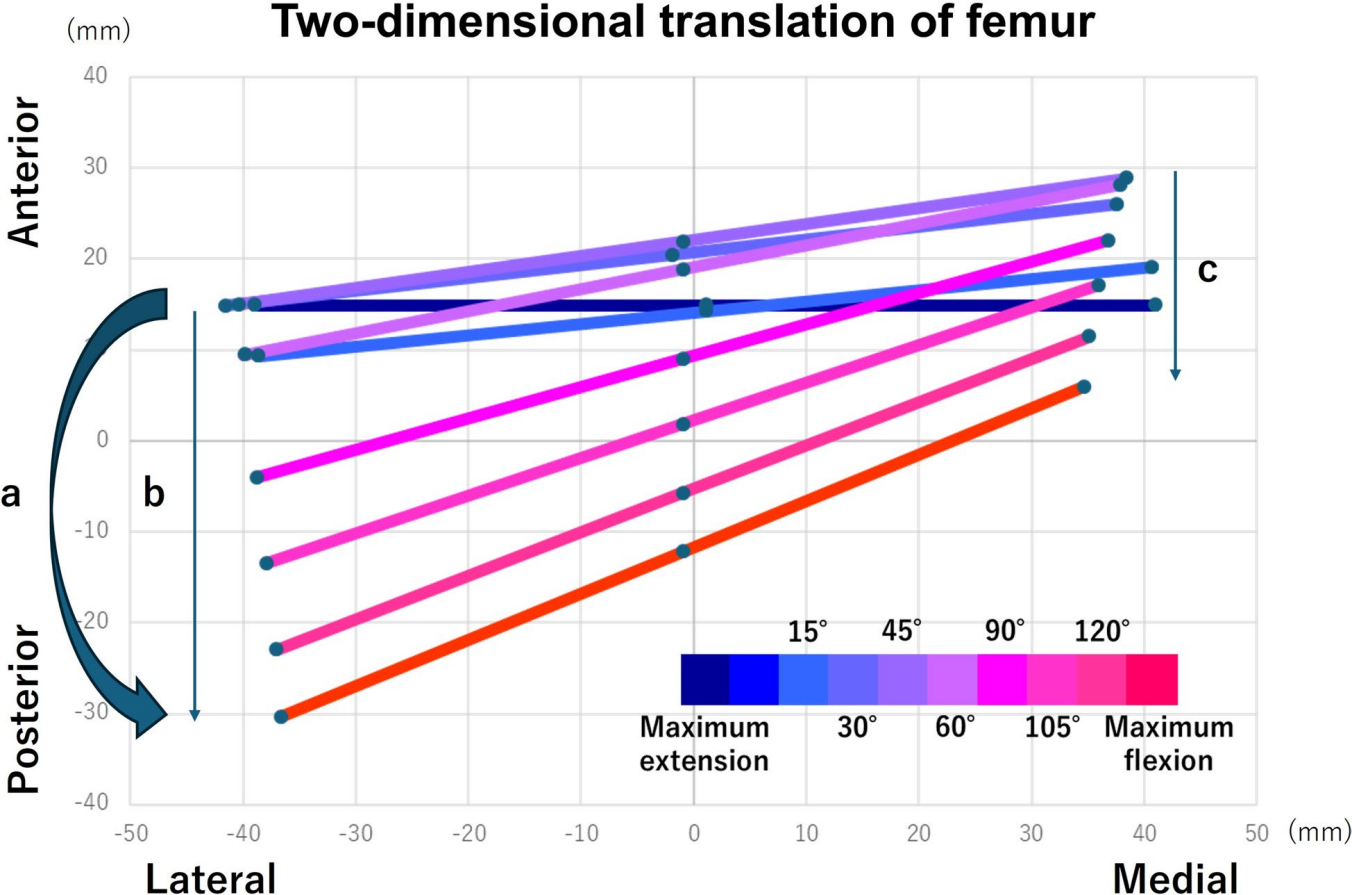
Two-dimensional translation of the femoral center relative to the tibia. **a** Temporal rotation relative to the tibia, defined as the difference in rotational angle between the flexion angle where the femur center was in its most anterior AP position relative to the tibia and the flexion angle where it was in its most posterior AP position. **b** AP translation at the lateral condyle, defined as the distance between the most anterior and posterior positions of the femur relative to the tibia at the lateral condyle. **c** AP translation at the medial condyle, defined as the distance between the most anterior and posterior positions of the femur relative to the tibia at the medial condyle. The horizontal axis represents the ML position of the femur relative to the tibia, and the vertical axis represents the AP position of the femur relative to the tibia. *AP* anteroposterior position of the femur relative to the tibia, *ML* mediolateral position of the femur relative to the tibia

Intraoperative knee kinematics were assessed using a previously reported method [1]. Two-dimensional translation of the femoral center was measured on the basis of data obtained from the navigation system, and knee kinematics were evaluated. The femoral axis at each measured flexion angle was projected onto the tibial axial plane to assess femoral motion relative to the tibia (Fig. 5). The axial diameter of the femur was set at 80 mm, and the position of the femur at each knee angle was calculated from the rotation angle of the tibia relative to the femur using the navigation system. Furthermore, we assessed the mediolateral position of the femur relative to the tibia in the same manner, projecting the femoral axis movement onto the tibial axial plane at each knee flexion angle. The medial point was regarded as the medial condyle and the lateral point as the lateral condyle (Fig. 5). On the basis of previous studies [5, 11], we classified knee kinematic patterns into medial pivot and non-medial pivot patterns. A medial pivot pattern was defined according to the following three criteria: (1) the femur exhibited external rotation during knee flexion, as indicated by an increase in the external rotational angle from the position where the femur was located most anteriorly relative to the tibia to the position of maximum flexion; (2) the anterior–posterior translation of the lateral femoral condyle was greater than that of the medial condyle; and (3) the center of axial femoral rotation, determined by the relative motion of the medial and lateral condyles, was located medially. Two senior orthopedic surgeons evaluated all the kinematic patterns. On the basis of their judgment, we determined the intraoperative knee kinematics. In cases of disagreement among raters, the final classification was determined by consensus.

### Subjects

This study focused on four parameters: the amount of femoral rotation relative to the tibia, AP translation in the medial and lateral condyle, and rotational knee kinematics. The femoral rotation relative to the tibia was defined as the difference in rotational angle between the flexion angle at which the femur was in its most anterior AP position and the flexion angle at which it reached its most posterior AP position (Fig. 5). AP translation was defined as the distance between the most anterior and posterior positions of the femur, as shown in Fig. 5. These measurements were performed separately for the medial and lateral compartments. In addition, changes in the rotational knee kinematics were evaluated for each case.

### Statistical analysis

Analyses were performed using SPSS Statistics (IBM SPSS Statistics, version 31; IBM Corp, Armonk, NY, USA). Given the limited sample size and the presence of repeated measures across implant conditions, nonparametric statistical methods were used. The Friedman test was applied to assess differences in the amount of rotation and AP translation among preoperative condition and the three implant designs. When a significant difference was identified, post hoc pairwise comparisons were conducted using Bonferroni correction to adjust for multiple comparisons. A power analysis was conducted on the basis of the mean and standard deviation calculated from three preliminary measurements to determine the required sample size for the primary outcome measures. For detecting a mean difference in rotational angle (*δ *= 4, *σ* = 5), the minimum required sample size was eight, assuming 80% power and a significance level of *α* = 0.05. For anterior–posterior translation (*δ* = 10, *σ* = 9), the required minimum sample size was nine under the same assumptions. Accordingly, we included nine specimens to satisfy both criteria. Statistical significance was set at *p* < 0.05. To assess interrater reliability for classification of knee kinematic patterns (Medial pivot and non-medial pivot pattern), Cohen’s kappa coefficient was calculated using SPSS Statistics, which indicated almost perfect agreement (*κ* = 0.944, *p* < 0.001).

## Results

**Fig. 6:**
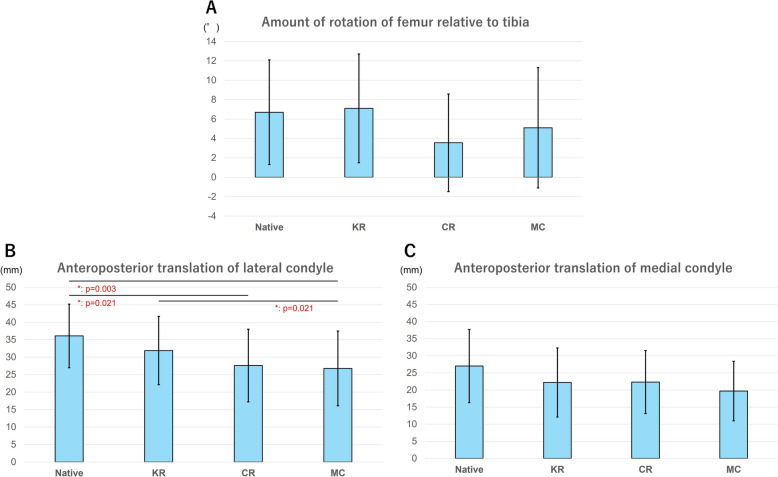
Changes in femoral rotation and anteroposterior translation during flexion among different TKA designs. **a** Rotational angle of the femur relative to the tibia during flexion in each group. The vertical line shows the rotational angle of the femur relative to the tibia (a positive value indicates external rotation of the femur relative to the tibia). **b** Anteroposterior translation of the lateral femoral condyle during flexion in each group. The vertical axis represents the anteroposterior translation of the femur relative to the tibia (a positive value indicates the posterior movement of the femur relative to the tibia). **C** Anteroposterior translation of the medial femoral condyle during flexion in each group. The vertical axis represents the anteroposterior translation of the femur relative to the tibia (a positive value indicates the posterior movement of the femur relative to the tibia). *Native* before total knee arthroplasty, *KR* kinematic-retaining total knee arthroplasty, *CR* cruciate-retaining total knee arthroplasty using a cruciate-retaining design insert, *MC* cruciate-retaining total knee arthroplasty using a medial congruent insert

Table 1 presents the maximum knee extension and flexion angles for each group. No significant differences were observed between the groups (Table 1). Figure 6 shows the changes in femoral rotational angle and AP translation. Table 2 shows the femoral rotational angle relative to the tibia and the AP translation of the femur relative to the tibia at the lateral and medial condyle in the preoperative, KR, CR, and MC groups. The KR group demonstrated the greatest mean femoral external rotation relative to the tibia throughout the range of motion among the groups; however, there were no statistically significant differences. The KR group exhibited a significantly greater AP translation at the lateral femoral condyle during flexion compared with the MC group (*p* = 0.021). In addition, the preoperative group showed a significantly higher AP translation at lateral condyle during flexion than the CR and MC groups (*p *= 0.003 and 0.021, respectively). There was no statistically significant difference between the preoperative and the KR groups in any knee status. Regarding kinematic patterns, medial pivot motion was observed in six of nine knees in the KR group, compared with three cases in the CR group and four cases in the MC group. The preoperative kinematic pattern was preserved after surgery in 88.8%, 77.7%, and 88.8% of patients in the KR, CR, and MC groups, respectively (Table 3).

**Table 1:** Knee extension and flexion angles for each status

	Native mean ± SD (range)	KR mean ± SD (range)	CR mean ± SD (range)	MC mean ± SD (range)
Knee extension angle (°)	−2.5 ± 4.1 (−9.5 to 3.0)	−1.1 ± 3.1 (−4.5 to 4.5)	−1.7 ± 4.6 (−6.5 to 5.5)	−1.7 ± 4.2 (−6.5 to 6)
Knee flexion angle (°)	126.9 ± 9.5 (105.0–135.0)	128.7 ± 4.4 (122.0–134.5)	131.9 ± 4.9 (123.5–138.0)	128.1 ± 4.5 (123.5–138.0)

*Native* before total knee arthroplasty, *KR* kinematic retaining, *CR* cruciate retaining, *MC* medial congruent, *SD* standard deviation

**Table 2:** Comparison of amount of rotation and anteroposterior translation among preoperative status and different implant designs using the Friedman test and Bonferroni-corrected pairwise comparisons

	Native mean ± SD (range)	KR mean ± SD (range)	CR mean ± SD (range)	MC mean ± SD (range)	*p*-value (Friedman test)	Post hoc Bonferroni-corrected pairwise comparisons
Amount of the rotation (°)	6.7 ± 5.4 (−5.0 to 17.0)	7.1 ± 5.6 (−4.0 to 15.0)	3.5 ± 5.0 (−5.5 to 13.0)	5.1 ± 6.2 (−4.5 to 15.5)	0.131	Not applicable
AP translation at lateral condyle (mm)	36.1 ± 9.1 (16.0–46.3)	31.9 ± 9.8 (14.2–41.8)	27.6 ± 10.4 (9.6–40.9)	26.8 ± 10.7 (8.0–40.3)	< 0.001^*^	Native versus CR (*p* = 0.021) Native versus MC (*p* = 0.003) KR versus MC (*p* = 0.021)
AP translation at medial condyle (mm)	27.0 ± 10.7 (5.1–40.7)	22.2 ± 10.1 (5.0–34.8)	22.3 ± 9.2 (6.3–30.9)	19.7 ± 8.7 (5.9–29.9)	0.269	Not applicable

*Native* before total knee arthroplasty, *KR* kinematic retaining, *CR* cruciate retaining, *MC* medial congruent, *SD* standard deviation, *AP* anteroposterior

**Table 3:** Rotational knee kinematics for each knee status

	Native	KR	CR	MC
Case 1	Medial pivot	Medial pivot	Medial pivot	Medial pivot
Case 2	Medial pivot	Medial pivot	Medial pivot	Medial pivot
Case 3	Medial pivot	Medial pivot	Non-medial pivot	Non-medial pivot
Case 4	Non-medial pivot	Non-medial pivot	Non-medial pivot	Non-medial pivot
Case 5	Medial pivot	Medial pivot	Non-medial pivot	Medial pivot
Case 6	Medial pivot	Medial pivot	Medial pivot	Medial pivot
Case 7	Non-medial pivot	Non-medial pivot	Non-medial pivot	Non-medial pivot
Case 8	Non-medial pivot	Medial pivot	Non-medial pivot	Non-medial pivot
Case 9	Non-medial pivot	Non-medial pivot	Non-medial pivot	Non-medial pivot

*Native* before total knee arthroplasty, *KR* kinematic retaining, *CR* cruciate retaining, *MC* medial congruent, *medial pivot* medial pivot pattern

## Discussion

The key finding of this study was that the implant design influences knee kinematics, and the KR design may facilitate more physiological kinematics, even when applied to OA knees. In this study, a kinematic evaluation was performed using a navigation system, which allowed for precise measurements of flexion angles throughout the full range of motion, enabling accuracy and consistency by employing the same coordinate system both preoperatively and postoperatively. The KR design exhibited a higher incidence of medial pivot motion and demonstrated significantly greater AP translation at lateral condyle than the MC designs. Although further investigation of the clinical outcomes is warranted, this novel implant design holds promise for improving patient satisfaction. To the best of our knowledge, this is the first study to investigate knee kinematics of the KR design in comparison with other implant designs using cadaveric models and a navigation system.

Advancements in surgical support technologies, such as robotic and navigation systems, along with improvements in implant materials, have ushered TKA into a new phase. Over the past few decades, numerous studies have focused on improving patient satisfaction, with particular emphasis on knee kinematics. The impact of kinematics on clinical outcomes has been highlighted in several studies. Nishio et al. demonstrated that intraoperative medial pivot patterns positively influenced clinical outcomes [5], while Alesi et al. reported that postoperative medial pivot motion during daily activities was associated with improved patient-reported outcomes [12]. In addition to rotational kinematics, femoral rollback is also an important factor. Kono et al. found that AP translation of the femur after TKA was correlated with the sports subscales of patient-reported outcome measures [13]. Recently, BCR-TKA, which aims to preserve both the ACL and PCL to restore native ligament balance and knee kinematics, has garnered increasing attention [14]. However, some reports have indicated that even with BCR-TKA, postoperative kinematics differ from those of the native knee [15, 16]. Moreover, the extent to which the ACL retains its normal function in TKA for severe OA remains unclear [8]. Hamada et al. used cadaveric models to evaluate changes in knee kinematics by comparing native knees with those having only femoral component replacement, BCR-TKA, and CR-TKA. They reported that even with BCR-TKA, replacing the tibial articular surface resulted in deviations from the kinematics of the native knee [17], thereby emphasizing the critical importance of tibial articular geometry. Given these circumstances, the development and validation of new implant designs remain essential for improving clinical outcomes. Furthermore, the direct comparison of the novel KR concept with other implant types using identical cadaveric knee specimens represents a key novelty of this study.

Several studies have examined the differences in clinical outcomes between implant designs. Mugnai et al. reported that even within guided-motion TKA, differences in bearing geometry and resulting kinematics can affect performance [18]. Similarly, Hino et al. found that within the same implant system, CR designs provided greater mid-flexion stability than posterior stabilized (PS) designs [19], underscoring the importance of implant geometry in determining postoperative joint mechanics. The KR design evaluated in this study features a varus-inclined tibial articular surface, such as other TKA systems that replicate the native medial inclination of the tibial plateau. Favorable outcomes with such designs have been attributed to their anatomical design [20]. Using cadaveric specimens, we compared the kinematics of the KR design with other implants. The KR design demonstrated a higher proportion of medial pivot motion than other designs, even exceeding that observed in preoperative knees with mild osteoarthritis. This suggests that its anatomical replication may promote more physiological movement. Further studies are needed to determine whether the medial pivot motion observed with the KR design translates into improved clinical outcomes and long-term implant performance. In addition, the KR group exhibited greater lateral condylar AP translation than the MC group, although this should not be considered superior. The more constrained geometry of the MC insert may behave differently under physiological, weight-bearing conditions.

Previous reports have shown that preoperative factors have a particularly strong influence on postoperative kinematics. Seito et al. demonstrated that preoperative varus deformity and rotational kinematics significantly affect postoperative kinematics [21]. Ueno et al. evaluated the presence or absence of medial pivot motion using a computed tomography-free navigation system and reported that 76.7% of knees retained similar rotational movement postoperatively compared with the preoperative motion [22]. Similarly, Tominaga et al. showed that the AP position of the femur throughout the range of motion strongly correlated with pre- and postoperative assessments, highlighting the importance of preoperative kinematics and its evaluation [2]. Furthermore, Hino et al., using a computed tomography-free navigation system, reported that in CR-TKA, coronal plane kinematics (varus–valgus motion) showed a stronger correlation between the pre- and postoperative states than in PS-TKA [23]. In this study, although the KR design revealed a higher proportion of medial pivot motion compared with the other designs, some specimens had already lost physiological kinematics preoperatively, likely owing to the progression of osteoarthritis. Among these, certain cases demonstrated restoration of medial pivot motion post implantation, whereas others did not. This variability suggests that the ability of the KR design to restore physiological kinematics may depend on the degree of preoperative deterioration and individual anatomical characteristics. Further research is warranted to identify which preoperative kinematic profiles may benefit most from specific implant designs, especially under different alignment philosophies. On the basis of these findings and the results of previous studies, implant selection should not be based solely on design characteristics but should also consider the preoperative kinematic profile of the patient. In addition, in this study, we used mechanical alignment. Long-term outcome studies assessing implants which have a varus-inclined tibial articular surface with patient-specific alignment concepts, such as kinematic alignment, remain lacking. Given the current clinical applicability of mechanical alignment, the present study employed this approach to evaluate the KR design. Nevertheless, since both preoperative kinematics and alignment concepts can influence postoperative knee kinematics, it is necessary to investigate how various combinations of implant design and alignment strategies affect clinical and functional outcomes.

This study had some limitations. First, the kinematic analysis was performed under non-weight-bearing conditions because assessments were conducted intraoperatively, potentially limiting the ability to detect functional differences related to insert geometry. While previous studies have indicated that knee kinematics generally follow similar patterns in both weight-bearing and non-weight-bearing states, except during the initial phase of movement [24], further studies are needed to confirm this. Furthermore, we did not assess preoperative conditions such as alignment, cartilage degeneration, or the integrity of the anterior and posterior cruciate ligaments, all of which could affect the AP positioning of the femur relative to the tibia and knee kinematics. Regarding the cadavers, we used Thiel-embalmed specimens rather than fresh-frozen cadavers. Previous studies have shown no significant differences between these two preservation methods in terms of anatomical and soft tissue characteristics [25, 26]. However, as this study was conducted using cadaveric specimens, the effects of muscular forces and ligament constraints present in living knees could not be assessed. In addition, ligament tension during extension and flexion was not quantified using a gap tensor. Although the surgical procedures were standardized and no cases showed excessive tightness, the absence of objective data on ligament strain limits the interpretation of implant-specific kinematic differences. Future studies should incorporate ligament tension assessments to provide a more comprehensive understanding. In particular, our study focused specifically on the changes in rotational alignment and AP movement following implantation in the same cadaveric specimens. Our findings provide insight into the kinematic characteristics of different implant designs using a cadaveric model, and they do not directly translate to functional outcomes in living patients. Further clinical investigations are needed to evaluate how these kinematic features manifest in vivo and whether they contribute to functional benefits in patients undergoing TKA. In this study, as mentioned above, we conducted evaluations solely under a mechanical alignment concept, and the influence of implant design under other alignment philosophies could not be assessed. Given that, owing to the difficulty of obtaining preoperative imaging in this cadaveric model, both the tibial posterior slope and femoral rotational angle were uniformly set at 3° for all specimens, which may have influenced the results. To ensure uniform conditions across all specimens, no additional soft tissue releases and additional bone resection were performed, and slight flexion contractures remained in some cases. Although in clinical practice such contractures might be addressed through additional bone resection or soft tissue release to optimize the range of motion, these interventions were not undertaken in the present study to maintain consistency for the purpose of implant design comparison. This is considered another limitation of our study. Finally, the relatively small sample size limited the generalizability of our results. Despite these limitations, this cadaveric study enabled a direct comparison of different implant designs under identical conditions within the same knee. While further clinical studies are needed to clarify the impact of implant-related kinematic differences and AP translation on clinical outcomes, our findings offer useful information for future implant design and surgical strategy.

## Conclusions

Using identical cadaveric specimens and navigation-based analysis, distinct kinematic profiles were identified for each implant design. Notably, the KR implant demonstrated kinematics approximating native knee motion; however, these findings remain preliminary and warrant further clinical validation.

## Supplementary Information

Supplementary material 1.

Supplementary material 2.

Supplementary material 3.

Supplementary material 4.

Supplementary material 5.

Supplementary material 6.

Supplementary material 7.

Supplementary material 8.

Supplementary material 9.

## Data Availability

Raw data were generated at Ehime University. Derived data supporting the findings of this study are available from the corresponding author Kazunori Hino on request.

## Abbreviations

ACL: Anterior cruciate ligament
AP: Anteroposterior
BCR: Bicruciate-retaining
BCS: Bicruciate-stabilized
CR: Cruciate-retaining
KOA: Knee osteoarthritis
KR: Kinematic-retaining
MC: Medial congruent
OA: Osteoarthritic
PCL: Posterior cruciate ligament
PS: Posterior stabilized
TKA: Total knee arthroplasty
