# Supplementary figures and images for: Effect of the kinematic retaining design on knee kinematics in total knee arthroplasty: A cadaveric study using a navigation system
Source: Knee Surg Relat Res. 2025 Sep 16;37:38. doi: 10.1186/s43019-025-00290-5 (PMC12442271; doi:10.1186/s43019-025-00290-5)

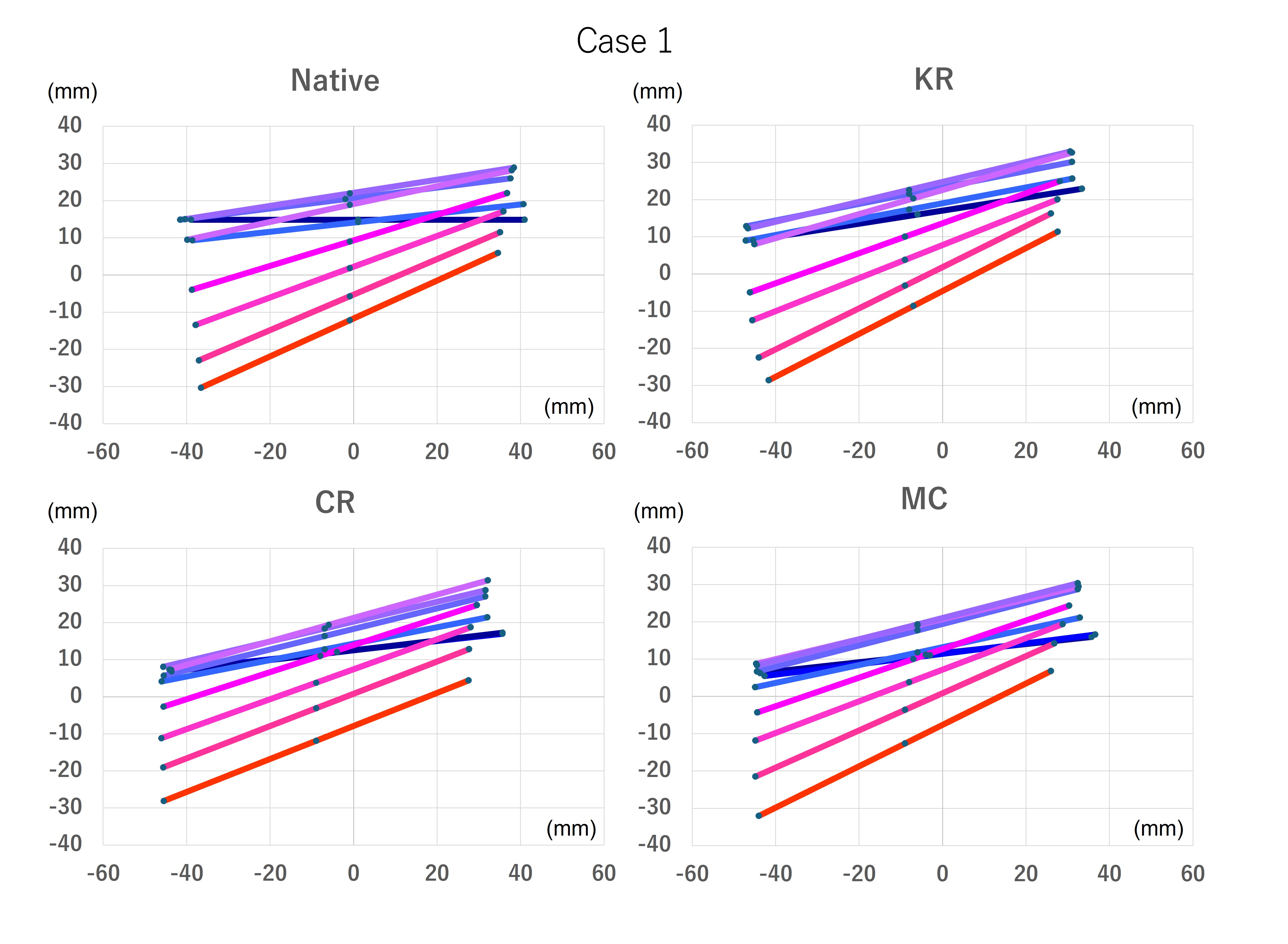

Supplement: Supplementary file 1 — Supplementary material 1. [file 43019_2025_290_MOESM1_ESM.jpg]

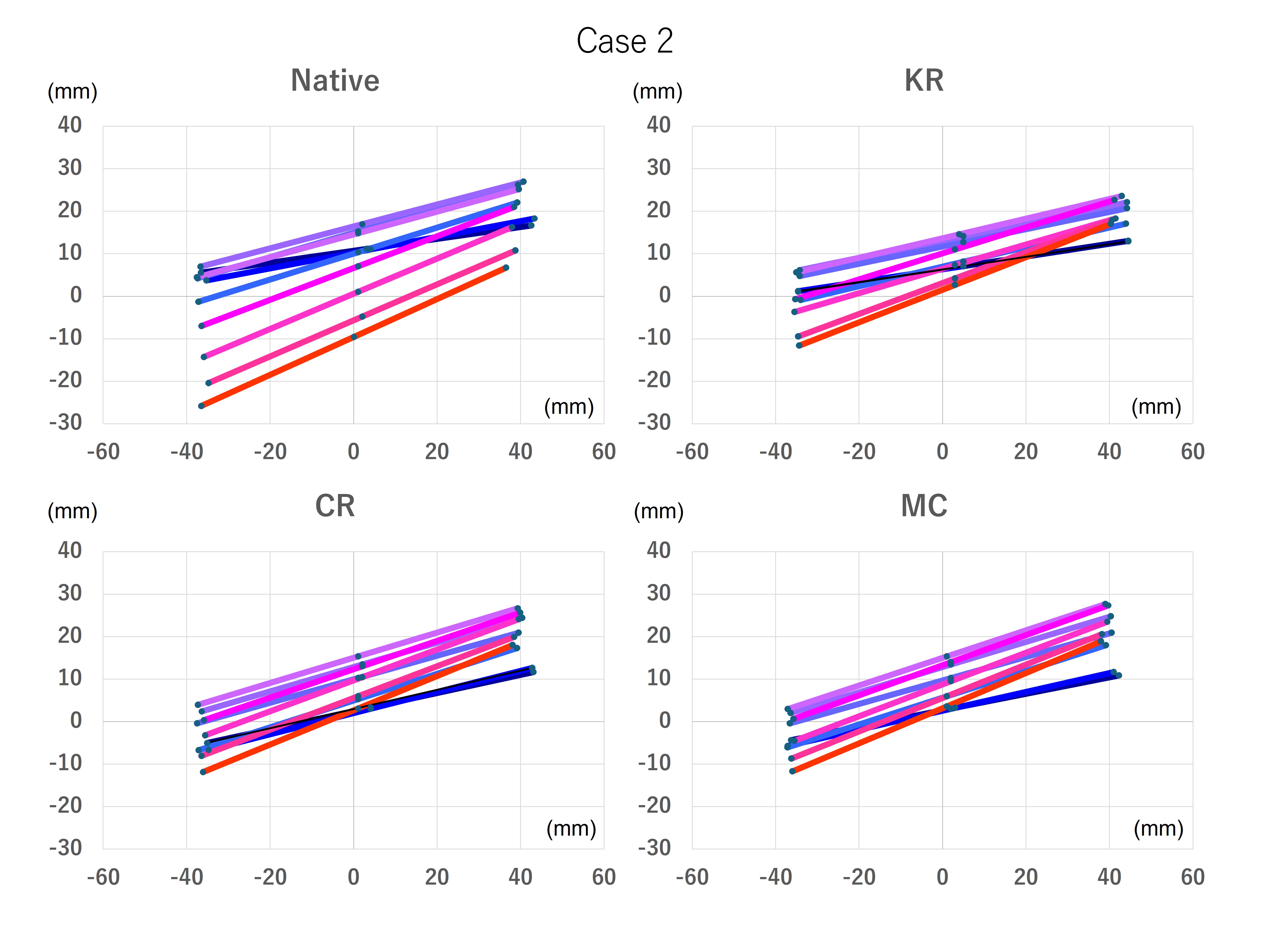

Supplement: Supplementary file 2 — Supplementary material 2. [file 43019_2025_290_MOESM2_ESM.jpg]

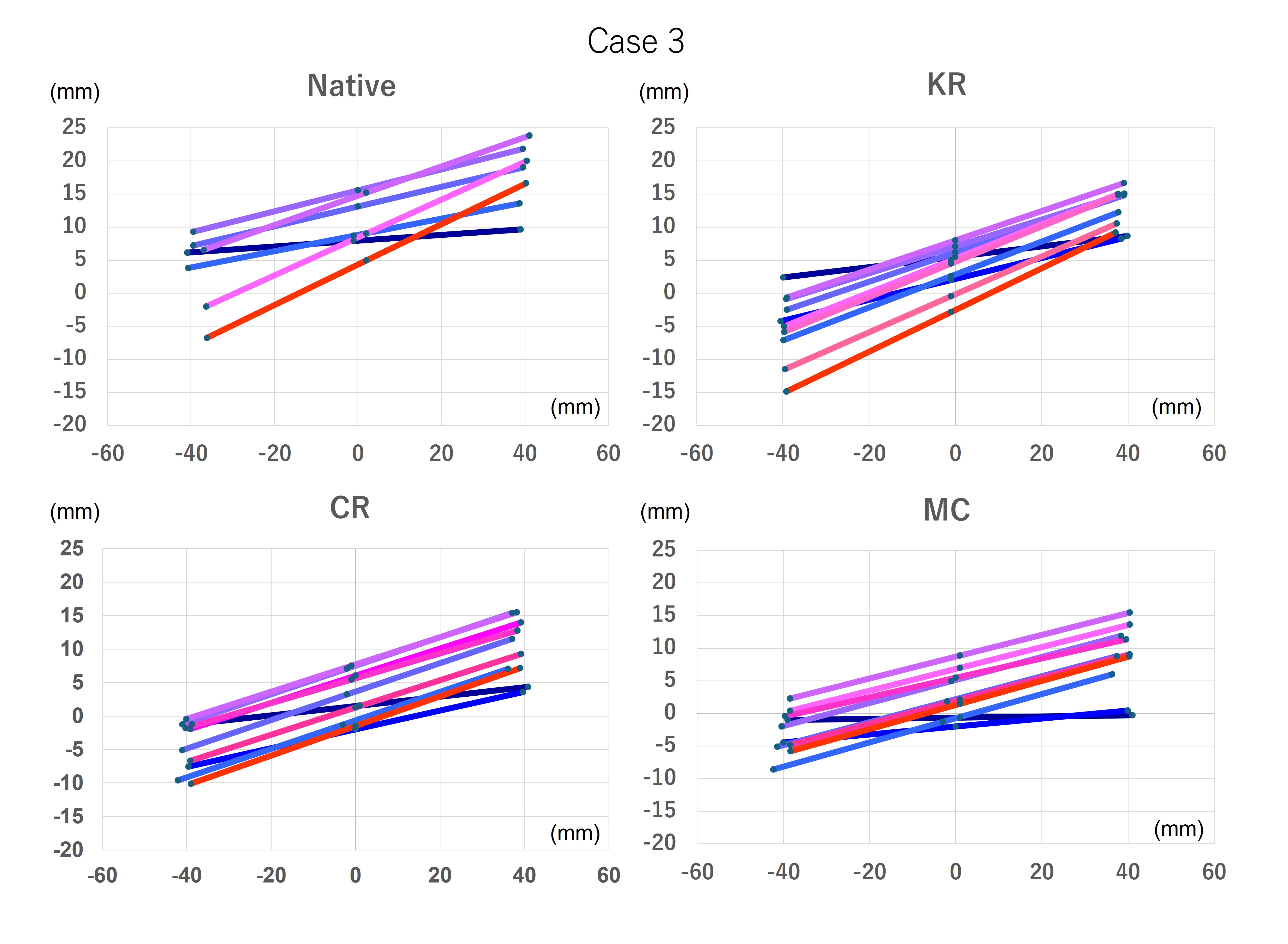

Supplement: Supplementary file 3 — Supplementary material 3. [file 43019_2025_290_MOESM3_ESM.jpg]

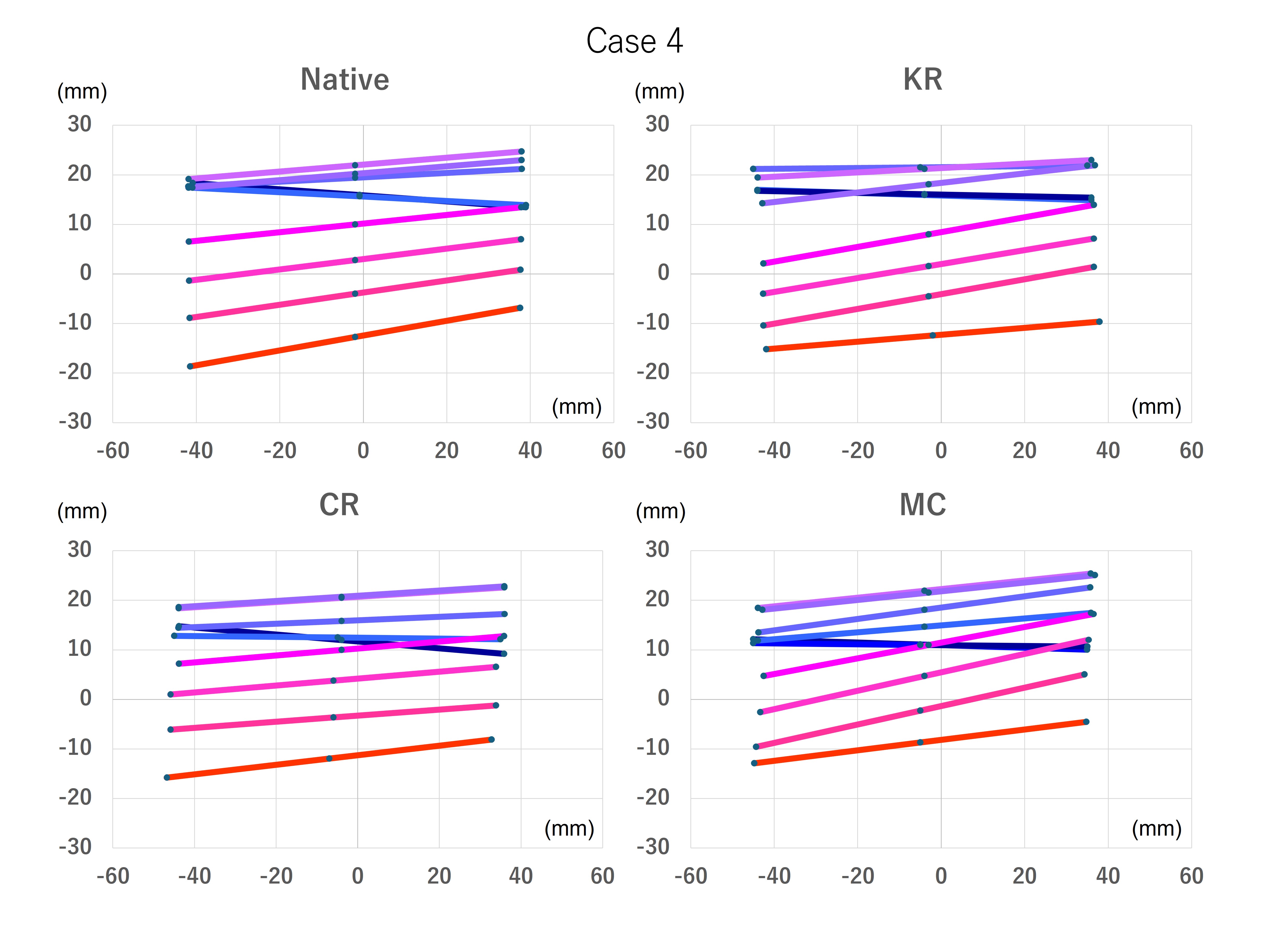

Supplement: Supplementary file 4 — Supplementary material 4. [file 43019_2025_290_MOESM4_ESM.jpg]

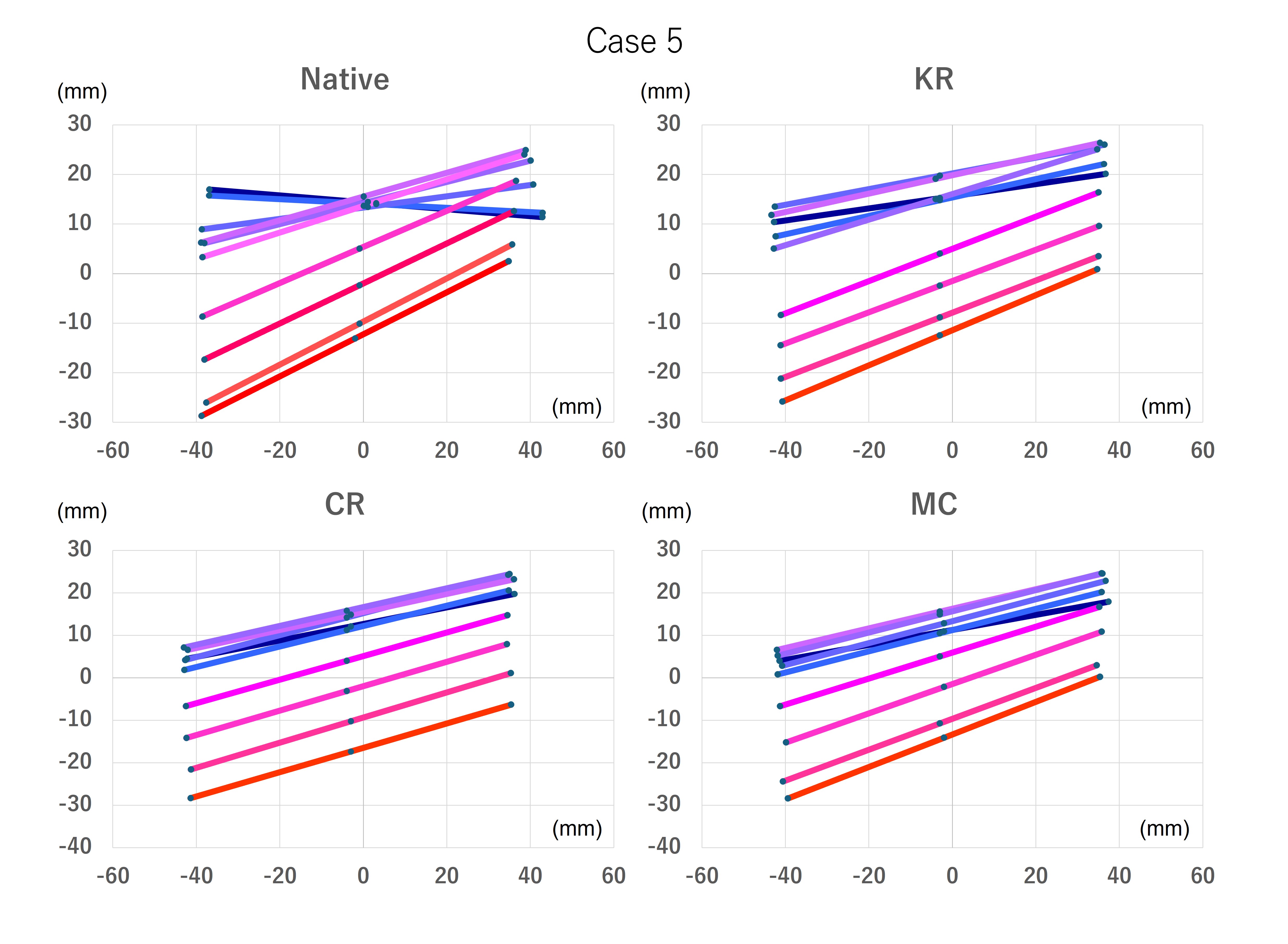

Supplement: Supplementary file 5 — Supplementary material 5. [file 43019_2025_290_MOESM5_ESM.jpg]

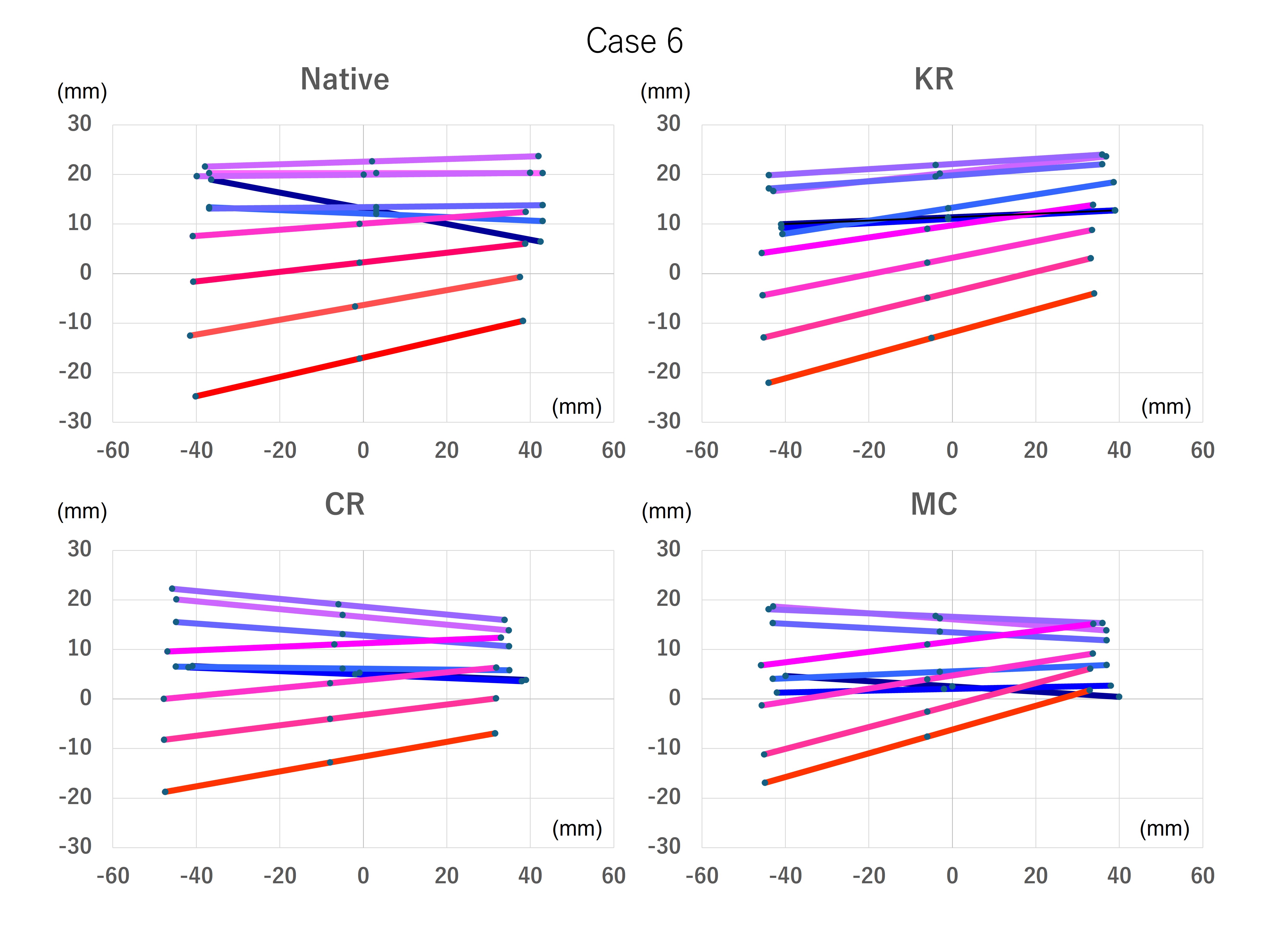

Supplement: Supplementary file 6 — Supplementary material 6. [file 43019_2025_290_MOESM6_ESM.jpg]

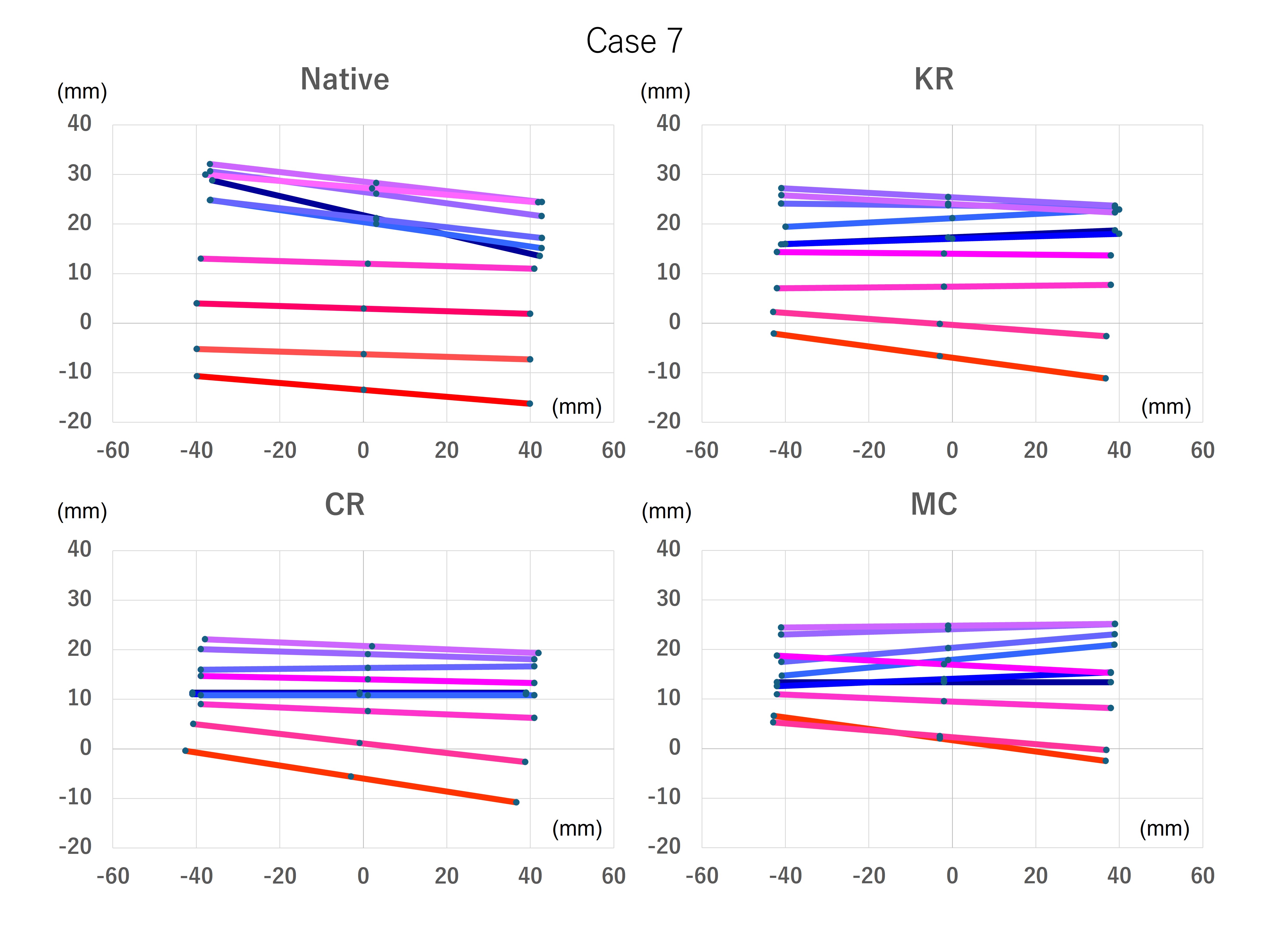

Supplement: Supplementary file 7 — Supplementary material 7. [file 43019_2025_290_MOESM7_ESM.jpg]

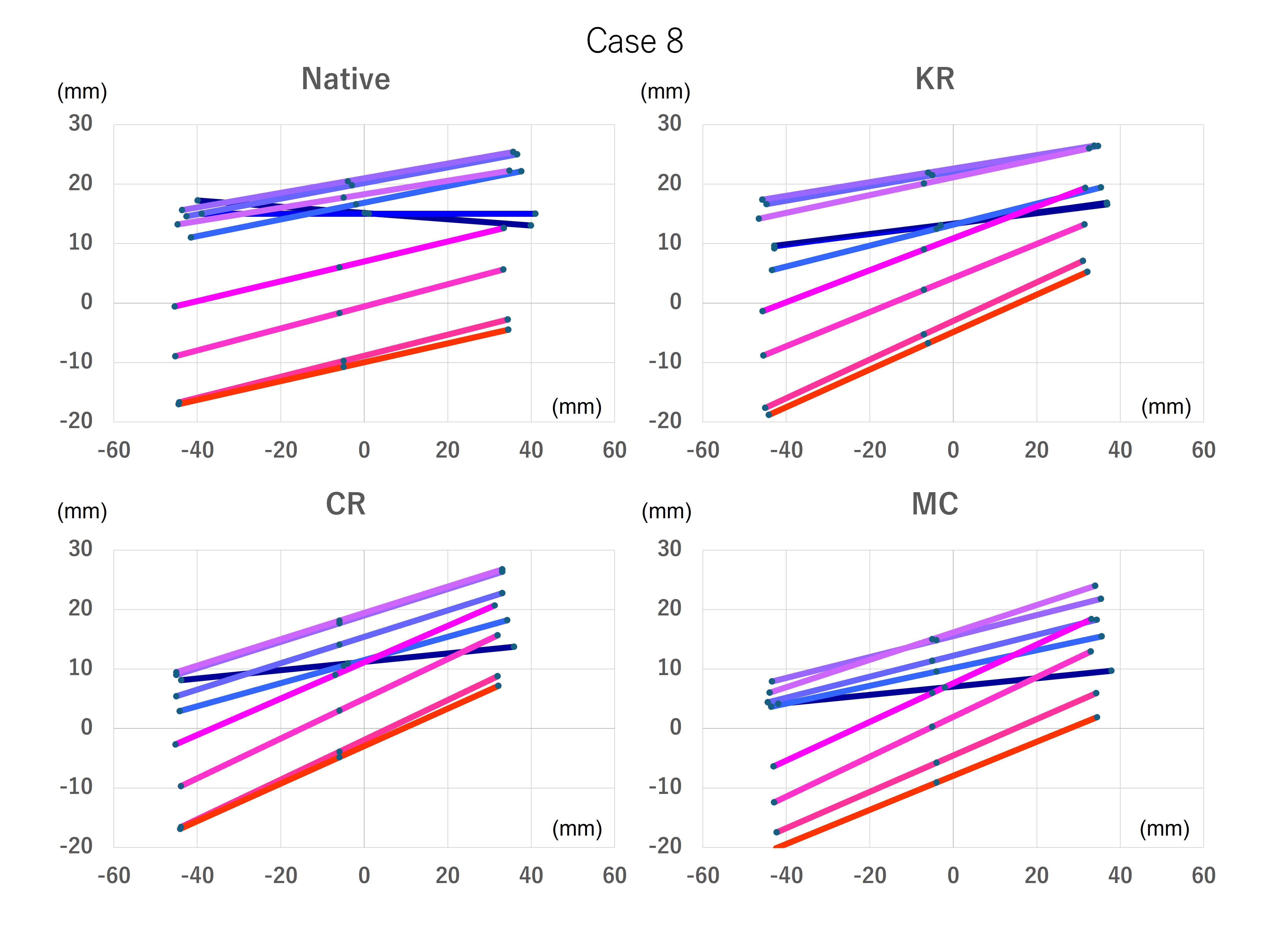

Supplement: Supplementary file 8 — Supplementary material 8. [file 43019_2025_290_MOESM8_ESM.jpg]

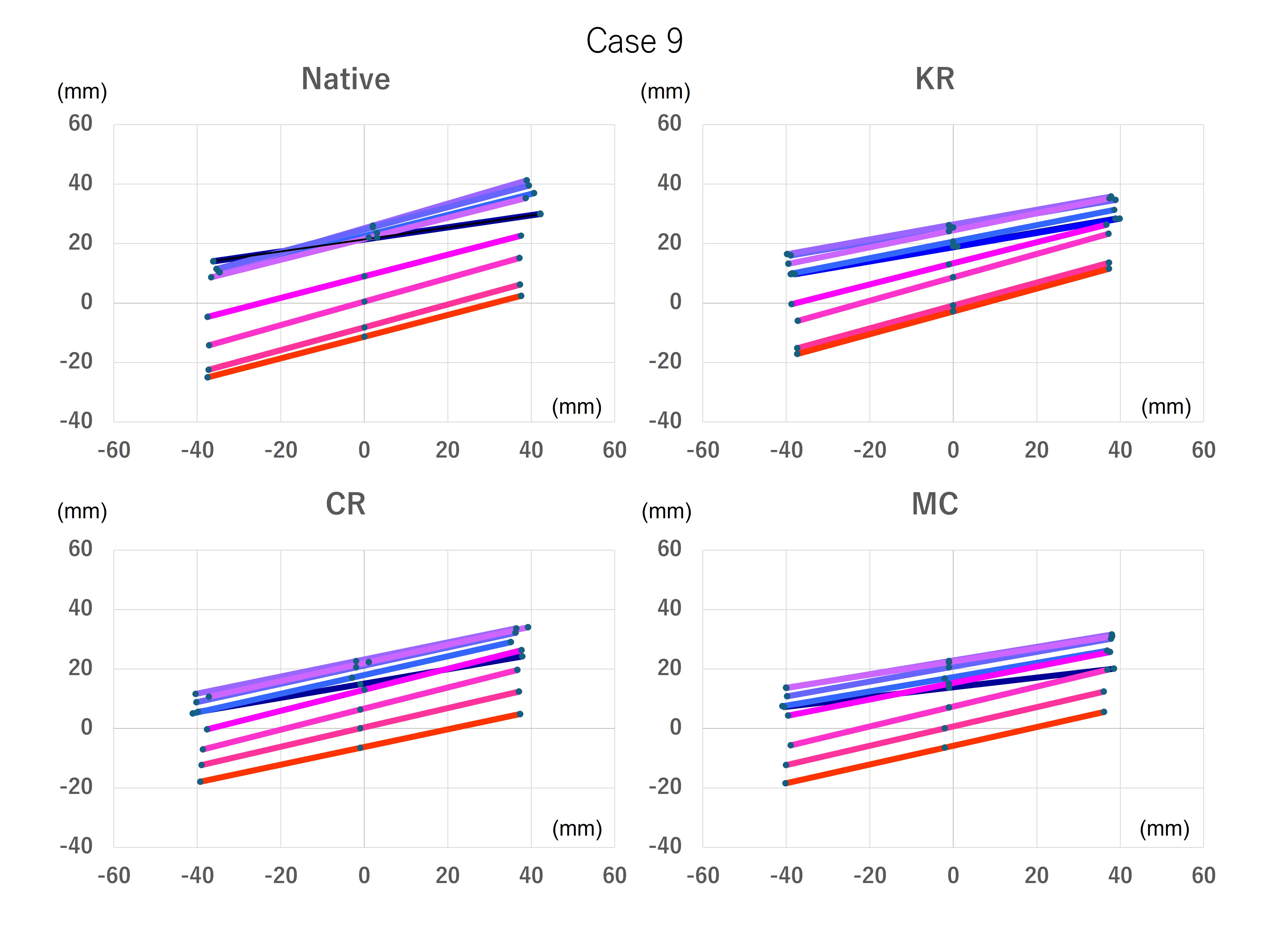

Supplement: Supplementary file 9 — Supplementary material 9. [file 43019_2025_290_MOESM9_ESM.jpg]
